# Experimental assessment of static and dynamic algorithms for gene regulation inference from time series expression data

**DOI:** 10.3389/fgene.2013.00303

**Published:** 2013-12-24

**Authors:** Miguel Lopes, Gianluca Bontempi

**Affiliations:** ^1^Machine Learning Group, Computer Science Department, Universite Libre de BruxellesBruxelles, Belgium; ^2^Interuniversity Institute of Bioinformatics in Brussels (IB)^2^Brussels, Belgium

**Keywords:** gene network inference, causality inference, temporal models, static models, experimental assessment

## Abstract

Accurate inference of causal gene regulatory networks from gene expression data is an open bioinformatics challenge. Gene interactions are dynamical processes and consequently we can expect that the effect of any regulation action occurs after a certain temporal lag. However such lag is unknown a priori and temporal aspects require specific inference algorithms. In this paper we aim to assess the impact of taking into consideration temporal aspects on the final accuracy of the inference procedure. In particular we will compare the accuracy of static algorithms, where no dynamic aspect is considered, to that of fixed lag and adaptive lag algorithms in three inference tasks from microarray expression data. Experimental results show that network inference algorithms that take dynamics into account perform consistently better than static ones, once the considered lags are properly chosen. However, no individual algorithm stands out in all three inference tasks, and the challenging nature of network inference tasks is evidenced, as a large number of the assessed algorithms does not perform better than random.

## 1. Introduction

The measurement of gene expression levels, by using microarrays or high throughput technologies, makes it possible to infer statistical dependencies (e.g., correlations) between the expression of two genes. Some of these dependencies can be seen as a result of causal interactions, as the expression of a gene can influence the future expression of another gene (these causal interactions are known as gene regulatory interactions). Several methods have been proposed to infer gene regulatory interactions from measured gene expression levels. Some of them are static, in the sense that they do not take temporal aspects into consideration, while others are designed in order to learn the dynamical aspects of the dependencies. Since gene interactions are not instantaneous, we expect that temporal aspects should shed light on the causal dependencies between genes. In other terms if two genes are part of a regulatory interaction, their expression levels over time are expected to be correlated with a certain lag and the time order is expected to elucidate the respective promoter/target roles. However, unfortunately such lag is unknown a priori and should be properly learned from data. If on one hand dynamic approaches may appear as more powerful than static ones because of the temporal representation, on the other hand they are more sensitive to the accuracy of the adopted lag. In machine learning jargon, this is known as a bias/variance trade-off. The adoption of temporal dynamic models makes the learner less biased but necessarily more exposed to high variance. In spite of this intuition, and although there are some comparisons between dynamic and static methods in the literature on gene regulatory networks, these are not systematic or extensive.

For this reason, we propose in this paper an experimental setting to assess the role of dynamics on the accuracy of the inferred regulatory network. To this aim, we compare a number of state-of-the-art static and dynamic approaches on three challenging inference tasks. As state-of-the-art static approaches, we consider Bayesian networks (Balov and Salzman, 2010; Kalisch et al., 2012) and directed graphical Gaussian models (GGM) (Schäfer and Strimmer, 2005). These two methods are based on the estimation of conditional dependencies between genes. The first infers a directed network using the rules of d-separation, the latter is an undirected graphical model (an edge indicates the presence of a conditional linear correlation between the respective nodes), but that can be made directed by making ad hoc assumptions. As dynamic approaches we consider: Vector AutoRegressive models (VAR) (Charbonnier et al., 2010), Dynamic Bayesian networks (DBN) (Lebre, 2009) and adaptive lag methods (Zoppoli et al., 2010; Lopes et al., 2012). VAR models are linear models where the target variable at a time point is modeled as a linear combination of predictor variables at previous time points (typically one). DBN are graphical models where variables at different time points are represented by different nodes and edges are allowed only from variables at time *t* to variables at time superior than *t*. Adaptive lag models are dynamic approaches which include an automatic estimation of a temporal lag for each pair of genes, e.g., by maximizing some dependence score. In order to make a fair comparison, all the assessed approaches (static and dynamic) are causal, in the sense that they infer directed interactions.

Our experimental study makes an assessment of static and dynamic algorithms by comparing the accuracy of the networks inferred from three microarray time series. These datasets have different characteristics, in terms of biological species, time length and sampling period (5, 10, and 30 min). The first outcome of the study is that dynamic models perform consistently better than static ones. The second outcome is an interesting insight on the most probable interaction lag between gene expressions. Our results suggest that this lag can take values in the range of a few hours, and that temporal network inference models should be adjusted to incorporate this information. In the next chapter we will present the assessed network inference algorithms, the third chapter describes the experimental setting and is followed by the results and discussion.

**Network Inference**

- Q: Which kinds of biological networks have been inferred in the paper?
- A: 500 gene regulatory networks of 5 nodes were inferred for three species (*E.coli*, yeast, fruit fly). Networks were inferred from time series gene expression datasets.
- Q: How was the quality/utility of the inferred networks assessed. How were these networks validated?
- A: The gold standard was defined as being interactions reported in the literature. A precision recall curve, and the respective area under (AUPRC) was assigned to each inferred network. The AUPRC values of the 500 networks predicted by an inference method were averaged, and this value was used to score that method.
- Q: What are the main results described in the paper?
- A: The general performance of state of the art network inference methods on the proposed task is weak (in two species, most of the methods do not have a performance significantly better than random). However, methods that take into account temporal information tend to perform better than static, non-temporal methods. The performance of temporal methods is expected to depend on the temporal sampling interval and on the sample size of the used time series. This fact is confirmed in our experiments and we infer general conclusions on the proper use of temporal network inference methods.

## 2. Materials and methods

Two family of network inference algorithms, static and dynamic, are considered in this study and will be discussed in the following section. Table 1 summarizes the differences between the used models.

**Table 1:** **Assessed network inference models**.

**Method**	**Type**	**Lags**	**Category**	**Features**
catnet	Static	–	Bayesian network	– Categorization of data
				– Stochastic search (simulated annealing) in the network space
*pcalg*	Static	–	Bayesian network	– Progressive removal of edges (backwards selection)
				– Conditional dependence estimated with partial correlation
*GeneNet*	Static	–	Graphical Gaussian Model	– Full partial correlations estimated through shrinkage
				– Edges are directed from the most to the less exogenous variable
*VAR I +lars*	Dynamic	Fixed (first)	VAR	–VAR(I) model subject to a LI penalty term
				– Regression coefficients estimated with least angle regression (lars)
*simone*	Dynamic	Fixed (first)	VAR	–VAR(I) model subject to a variable penalty term (to favor the selection of transcription factors)
				– Regression coefficients estimated through optimization
*GI DBN*	Dynamic	Fixed(first)	Dynamic Bayesian network	– Estimation of a number of first order partial regression coefficients, for each possible interaction
				– Predictors and target are lagged by I time point
*Time Delay ARACNE*	Dynamic	Estimated(one)	Information–theoretic	– Mutual information used to infer dependencies (MI estimated with a copula–based approach)
				– Estimation of the lag between two genes
				– Use of the DPI to break up fully connected triplets
*Time lagged MRNET*	Dynamic	Estimated(one)	Information–theoretic	– Mutual information used to infer dependencies (Gaussian assumption)
				– Estimation of the lag between two genes
				– mRMR feature selection
*Time lagged CLR*	Dynamic	Estimated(one)	Information–theoretic	– Mutual information used to infer dependencies (Gaussian assumption)
				– Estimation of the lag between two genes
				– Normalization of MI

### 2.1. Static models

Static network inference models do not take into account any information related to the temporal nature of the gene expression data. Two well-known examples are Bayesian networks and GGM.

A Bayesian network is a graphical representation by directed acyclic graph of a multivariate probability distribution, where nodes denote variables and edges variable dependencies. Under the faithfulness assumption for the probability distribution, there exists a bijective mapping between the conditional independencies of variables in the distribution and topological properties (d-separation) in the graph. The main advantages of a Bayesian Network representation are its sparsity (i.e., use of few parameters), the ease of interpretation and the availability of several inference algorithms. For further references on the estimation of Bayesian networks from biological data see Needham et al. (2007) or Margaritis (2003).

A GGM is an undirected graph, where the presence of an edge indicates a non zero partial correlation between two nodes given all the others (Dempster, 1972; Lauritzen, 1996). Partial correlations can be obtained by inverting the covariance matrix, but this is problematic if the covariance matrix does not have full rank. One solution is a positive definitive estimation of the covariance matrix (Opgen-Rhein and Strimmer, 2007). Another approach estimates partial correlations using the eigenvectors of the covariance matrix associated with non-zero eigenvalues (Lezon et al., 2006). It has been shown that partial correlations emerge, under the assumption that the variables are Gaussian-distributed, when maximizing the entropy of the system conditioned on the empirical mean and covariance of the variables (Lezon et al., 2006). Below we describe three implementations of static models, available in R packages: two estimations of Bayesian networks and one estimation of a GGM with an extension to direct some of its edges.

The R package *catnet* (Balov and Salzman, 2010) infers categorical Bayesian networks from categorical data (the variables have to be discrete, taking only a finite number of values). The maximum likelihood criterion is used to assess different possible networks. This package implements a stochastic search in the network space, using a simulated annealing algorithm. In the experiments here presented, we defined the number of categories to be three (corresponding to different levels of gene expression). The output of this algorithm is a number of networks (represented by adjacency matrices) of increasing complexity each annotated with a likelihood. In order to obtain a final score matrix we made a weighted sum (based on likelihood) of all adjacency matrices.

The package *pcalg* (Kalisch et al., 2012) infers Bayesian networks from continuous data, and is based on the PC algorithm (Spirtes et al., 1993). The PC algorithm starts by considering a fully connected graph and progressively removes edges, depending on the conditional dependencies between the respective genes. The size of the conditioning sets is one at the beginning and then gradually increased. The existence and the direction of the edges is inferred using the rules of d-separation. In our experiments, the conditional dependence is measured by partial correlation, which is equivalent to assume that the variables are Gaussian distributed and their dependencies linear. The Fisher transformation is used to compute the significance level of the partial correlation value. By defining a set of decreasing threshold values for the significance level, we obtained a number of inferred networks with an increasing number of edges. Then we associated to each possible interaction a score equal to the average number of times that this interaction is inferred in the returned networks.

*GeneNet* (Opgen-Rhein and Strimmer, 2007) estimates partially directed GGM. Once the positive definitive covariance matrix is estimated (using a shrinkage technique Schaefer et al., 2006), it computes the concentration matrix (the inverse of the covariance matrix) and a partial correlations matrix. An undirected GGM is created by selecting the edges associated to the highest partial correlations. GeneNet infers the directionality of the interactions by comparing, for each pair of connected nodes, the partial variances of the respective variables. The partial variance of a variable is its variation that cannot be modeled, or predicted, in a linear regression model using the other variables in the set as predictors. The ratio between the partial variance and the variance gives the percentage of the variation that corresponds to unexplained variation. These relative values of unexplained variation are used as indicators of how much of the variable variation can be explained from within the system (using all the other variables). An edge between two nodes is directed from the one with higher unexplained variation to the one with lower. Each edge is given a *p*-value (the null hypothesis is that the partial correlation between its nodes, or genes, is zero). For each edge we assigned a score equal to 1 minus the respective *p*-value.

### 2.2. Dynamic models

We will distinguish dynamic models according to the approach used to define the lag between variables. In what follows *p* is the number of genes and *X*^*t*^ is used to denote the value of the variable *X* at time *t*.

#### 2.2.1. Fixed lag models

Vector autoregressive models of order *l*_max_ (VAR(*l*_max_)) models each gene *X*^*t*^, at time *t*, as a linear function of all the genes at time *t* − *l*, where *l* = 1,.., *l*_max_.

(1) $$X_{i}^{t}=c+\sum_{l=1}^{l_{\text{max}}}\sum_{j=1}^{p}\beta_{l,j}X_{j}^{t-l}+\epsilon_{i}$$

Therefore VAR(1) denotes a lag-one model where the value of *l*_max_ is set to 1. The coefficients β in (1) can be estimated by Ordinary Least Squares algorithm (OLS), provided that there are enough samples. Alternatively, β can be returned by a regularization algorithm, such as the *Lasso* (Tibshirani, 1994), which adds a penalty term in the OLS solution equation, that is proportional to the *L*_1_ norm of β. In other words, the Lasso minimizes the sum of squares of the residuals, given that the sum of the absolute value of the coefficients β is less than a constant. This approach imposes scarcity in the number of returned non-zero coefficients and can be used to detect the most relevant coefficients.

Another fixed lag model is the Dynamic Bayesian Network (DBN). DBN are modifications of Bayesian networks to model time series: each gene is represented by different nodes, at different time points (Perrin et al., 2003). An edge is allowed to go from a node *X*^*t* − *l*^ to a node *Y*^*t*^. In our study we assessed three lag-one models, two of them penalty-constrained implementations of VAR(1) models, and one of them an implementation of a DBN. They are described below.

Our implementation *VAR(1) + lars* models the data from a VAR(1) perspective: a variable *X*^*t*^_*i*_ is regressed using all the variables lagged by one time point: *X*^*t* − 1^_*j*_, *j* = 1 … *p*. As with the Lasso, a penalty term proportional to the *L*_1_ norm of the regressor coefficients is added to the model. The coefficients of the model are estimated using the *lars* algorithm [(Efron et al., 2004), available in the R package *lars*]. The lars algorithm computes in a fast manner the coefficients of the lasso path, when the regularization penalty term goes from infinity (where there is no non-zero returned coefficients) to 0 (corresponding to the OLS solution). Using lars, for each gene we computed the coefficients of its predictors at the points (in the lasso path) where the coefficient of a predictor becomes non-zero and enters the model. We then computed the average of the coefficients of each predictor variable and used it as the directed dependence score between the predictor and the target gene.

The R package *simone* (Charbonnier et al., 2010) estimates the coefficients of a VAR1 model subject to a *L*_1_ norm penalty term. Here, a weighted lasso is used, a modification of the Lasso to allow different penalty terms for different regressors. Genes are grouped into two main groups: *hubs*, which are genes that show a high level of connectivity probability to all the other genes, and *leaves*, which are only connected to hubs. It is suggested that hubs will correspond to transcription factors (genes whose expression levels influence the transcription of other genes). Every gene is assigned to the group of hubs or to group of leaves, from an initial estimation (or optionally, from expert knowledge if available). This initial estimation is done by computing a matrix of coefficients using the standard Lasso, and then group genes into hubs or leaves according to the *L*_1_ norm of the respective rows in the estimated coefficients matrix. The regressors are assigned one of two different weights, one for hubs and the other for leaves, which multiply the respective coefficients before they are used in the calculation of the penalty term. The idea behind this implementation is that interactions coming from hubs (transcription factors) should be less penalized than interactions coming from leaves. Simone returns a list of inferred networks for different values of the penalty weights. In the experiments here reported, we defined the score for an interaction as the number of times the interaction is associated with a non-zero coefficient in all the returned networks.

*G1DBN* is a R package (Lebre, 2009) that estimates dynamic Bayesian networks, using first order conditional dependencies. G1DBN is designed to work with time series and implements a lag-one model. Each gene is represented by two nodes lagged by one time point. Interactions are only allowed from nodes at time *t* − 1 to nodes at time *t*. It is a two-step method: the first step computes all possible regression coefficients, of each gene *X*^*t* − 1^_*j*_ to each gene *X*^*t*^_*i*_, conditioned on each other gene *X*^*t*^_*k*_, *k* ≠ *j, i*. This way, each directed interaction is assigned a number of coefficients, one for each conditioning variable. Each of these coefficients is subject to a statistical test based on the student's t distribution (the null hypothesis is that the value is zero) and a *p*-value is returned. The maximum of these *p*-values is considered as a score for the respective interaction. A threshold α_1_ is defined, and edges with scores lower than it are selected. The second step of the algorithm starts with this graph and removes more edges: for each gene, it is calculated the regression coefficient of it toward one of its parents, given all the other parents. To each of these coefficients is assigned a *p*-value, in an analogous way as in the first step. A new threshold α_2_ is defined, and only edges with *p*-values lower than α_2_ are kept. In our experiments, we defined α_1_ = 0.7, as it was the value used in the method's original proposal. We used several values for α_2_, and for each of them an adjacency matrix was returned, with the estimated *p*-values for each possible interaction. For each interaction, the subtraction 1 minus the average of the respective final *p*-values, was used as the final score.

#### 2.2.2. Adaptive lag models

Adaptive lag models are models where each possible interaction is assigned a score which is a function of an estimated temporal lag, that hypothetically characterizes the interaction. This lag is estimated as the one which maximizes some score *S*. The lag between two genes *X* and *Y* is estimated as:

(2) $${\text{lag}}^{XY}=\arg\underset{l}{\max}\left(S\left(X^{t},Y^{t-l}\right)\right)l=-l_{\text{max}},..,-1,0,1,..,l_{\text{max}}$$

The parameter *l*_max_ is the maximum allowed lag. The adaptive lag methods implemented are based on the measure of mutual information (which is represented as *I*(*X; Y*), between two variables *X* and *Y*).

The *Time-Delay ARACNE* (Zoppoli et al., 2010) is an extension of the information theoretic algorithm ARACNE (Margolin et al., 2005). It is based on three steps: the first step estimates the times at which each gene starts to be differentially expressed (and the set of possible interactions is restricted to the directed interactions where the target gene has a start-of-regulation time higher than the start-of-regulation time of the source gene). The second step of the algorithm lags the temporal expression of each pair of genes, and finds the lag which maximizes the mutual information between the genes. The mutual information is estimated through a copula based approach. A copula transformation (a rank based empirical copula) is applied to the distribution, and a kernel density estimator is used to estimate the bivariate marginal distribution $\hat{p}(X_{i}^{t},X_{j}^{t-l})$, for each gene *X*^*t*^_*i*_ and each gene *X*^*t* − *l*^_*j*_. The directed edges whose lagged mutual information is higher than a defined threshold are kept in the graph. The third and final step of the algorithm applies the data processing inequality (DPI) property to break up fully connected triplets. A binary adjacency matrix, indicating the predicted interactions, is returned. We defined various values for the threshold and obtained different adjacency matrices. Each interaction is assigned a score equal to the number of times the interaction has been predicted in the returned adjacency matrices. The parameter *l*_max_ was set to 6 time points.

The *Time-lagged MRNET* is the dynamic extension of the MRNET algorithm (Meyer et al., 2007) which is based on the *minimum-Redundancy Maximum-Relevance* (mRMR) feature selection method (Ding and Peng, 2005). For each gene *Y*, it selects all other genes in a sequential manner. The first selected gene (added to the group of selected genes *S*) is the one that has the highest mutual information toward the target gene. The next gene to be selected, *X*^mRMR^_*j*_, is defined as the one which maximizes the following mRMR score, *u* − *r*:

(3) $$X_{j}^{\text{mRMR}}=\arg\underset{X_{j}\notin S}{\max}\left(u_{j}-r_{j}\right)$$

where *u*_*j*_ and *r*_*j*_ are defined as follows:

(4) $$u_{j}=I(X_{j};Y)$$

(5) $$r_{j}=\frac{1}{|S|}\sum_{X_{k}\in S}I\left(X_{j},X_{k}\right)$$

The term *u*_*j*_ represents the relevance of *X*_*j*_ toward *Y* and the term *r*_*j*_ represents the redundancy of *X*_*j*_ with the previously selected genes in *S*. This process is repeated for all genes. To any pair of genes the MRNET algorithm assigns a score which is equal to the maximum between two mRMR scores: the mRMR score of the first when the second is the target, and the mRMR score of the second when the first is the target. The time-lagged MRNET is a modification of the MRNET algorithm (Lopes et al., 2012). Here, the mutual information considered by the algorithm (between each pair of genes) is a lagged mutual information. The lag is the one which maximizes the mutual information, as in the Equation (2). The estimation of lags allows to direct interactions, as the sign of the lags provide information on the direction of interactions. Therefore, the time-lagged MRNET returns directed interactions, as opposed to the standard undirected MRNET.

The *Time-lagged CLR* is the dynamic version of the *context likelihood of relatedness* (CLR) inference algorithm (Faith et al., 2007). CLR takes into account the fact that some genes exhibit, on average, a relatively high, or low, mutual information toward all the other genes. Each possible interaction between *X* and *Y* is assigned a score equal to $w_{xy}=\sqrt{z_{x}^{2}+z_{y}^{2}}$, where:

(6) $$z_{x}=\max\left(0,\frac{I(X,Y)-\mu_{x}}{\sigma_{x}}\right)$$

μ_*x*_ and σ_*x*_ are the empirical mean and the standard deviation of the mutual information between X and all the other genes. This way, the CLR score for an interaction between genes *X* and *Y* is higher for situations when both *X* and *Y*, or any of them, exhibit a low mutual information toward the majority of remaining genes in the dataset, compared with the otherwise situation. The time-lagged CLR is a modification of CLR (Lopes et al., 2012), just as the time-lagged MRNET is relative to MRNET.

On the implementations here described, the mutual information used by the time-lagged MRNET and CLR was estimated with the Pearson correlation. The value for the maximum allowed lag parameter, *l*_max_, was set to be 6, 12, and 18 time points. In the following results, the time-lagged MRNET and CLR of a certain *l*_max_ are referred as TL *l*_max_ MRNET and TL *l*_max_ CLR, respectively (e.g., TL12 MRNET).

We note that the assessment here presented does not constitute an extensive review of all the causal network inference models found in the literature. These include dynamic models based on ordinary differential Equations, such as the *Inferelator* (Bonneau et al., 2006) or the *TSNI* (Bansal et al., 2006), and other implementations of Bayesian and Dynamic Bayesian networks, such as *Banjo* (Smith et al., 2006).

### 2.3. The datasets

Three time series datasets, from different species were collected. All these datasets are available in the Gene Expression Omnibus (GEO) database repository.

- A time series dataset of the gene expression of *Drosophila melanogaster*, of length 22 h (Hooper et al., 2007). The number of observations is 28 and the time between observations is 1 h after the 10 first observations, and approximately 30 min in the first 10 observations. We will refer to this dataset as *dataset Fly*.
- A time series dataset of the gene expression of *Escherichia coli*, of length 5 h and 20 min (Traxler et al., 2006). The number of observations is 17 and the time between observations changes between 10 and 50 min. We will refer to this dataset as *dataset E.coli*.
- A time series dataset of the gene expression of *Saccharomyces cerevisiae*, of length 2 h (Pramila et al., 2006). The number of observations is 25 and the time between observations is 5 min. We will refer to this dataset as *dataset Yeast*. This dataset is composed of two time series, and we averaged the samples of equal time points.

In the datasets Fly and *E.coli* we interpolated linearly the data, to obtain time series with a constant step: 30 min in the first and 10 min in the second. After this operation, the dataset *E.coli* has 32 time points and the dataset Fly has 45 time points.

### 2.4. Performance assessment

**Figure 1:**
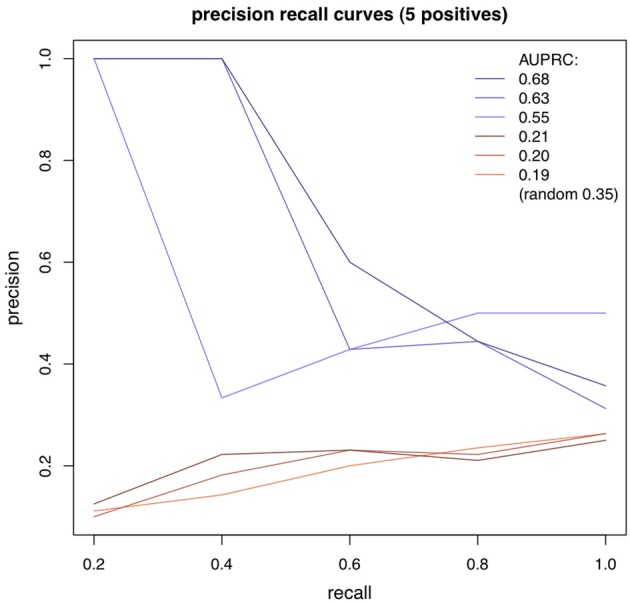
**Precision-recall curves**.

Adjacency matrices with documented interactions for the three different species were obtained in Gallo et al. (2010), Gama-Castro et al. (2011) and Abdulrehman et al. (2011) (for the Fly, *E.coli* and Yeast datasets). Only strong evidence interactions were selected. From these adjacency matrices, we generated small regulatory networks, containing only genes whose expression levels are measured in the respective dataset. For each dataset, 500 sub-networks of 5 nodes were randomly generated. Using the algorithms in the way that was described in the previous section, we obtained for each algorithm and network, a square matrix of scores for all possible directed interactions (the element (*i, j*) represents the score of the interaction from gene i to gene j). For any pair of genes, only one interaction was kept, corresponding to the strongest direction. To assess the performance of an algorithm on a given network we used the AUPRC (area under the precision recall curve). Interactions were incrementally selected (from the highest to the lowest ranked), and at each selection, precision and recall values were computed. We assigned to each recall its highest associated precision (there can be multiple precision values for a given recall). The AUPRC was estimated as the average precision, for all values of recall. For each algorithm and dataset, we averaged the AUPRC obtained for the 500 networks. The random baseline was estimated as being the expected average AUPRC of a random ranking, on all networks. Figure 1 shows some examples of precision recall curves, in blue with an higher AUPRC than the expected random baseline, and in red with lower (the number of instances is 20, and the number of positives is 5).

## 3. Results

**Figure 2:**
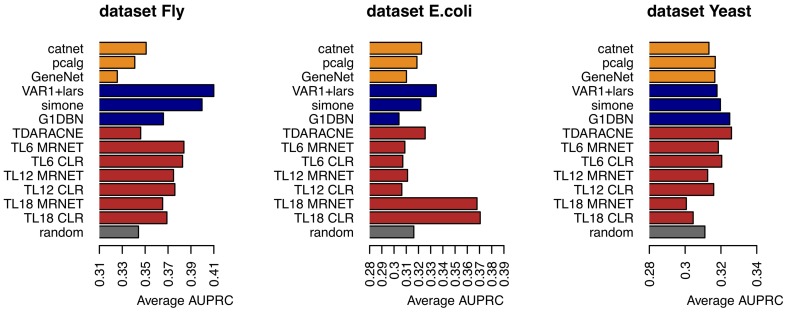
**Average AUPRC for the three datasets and different algorithms**.

**Figure 3:**
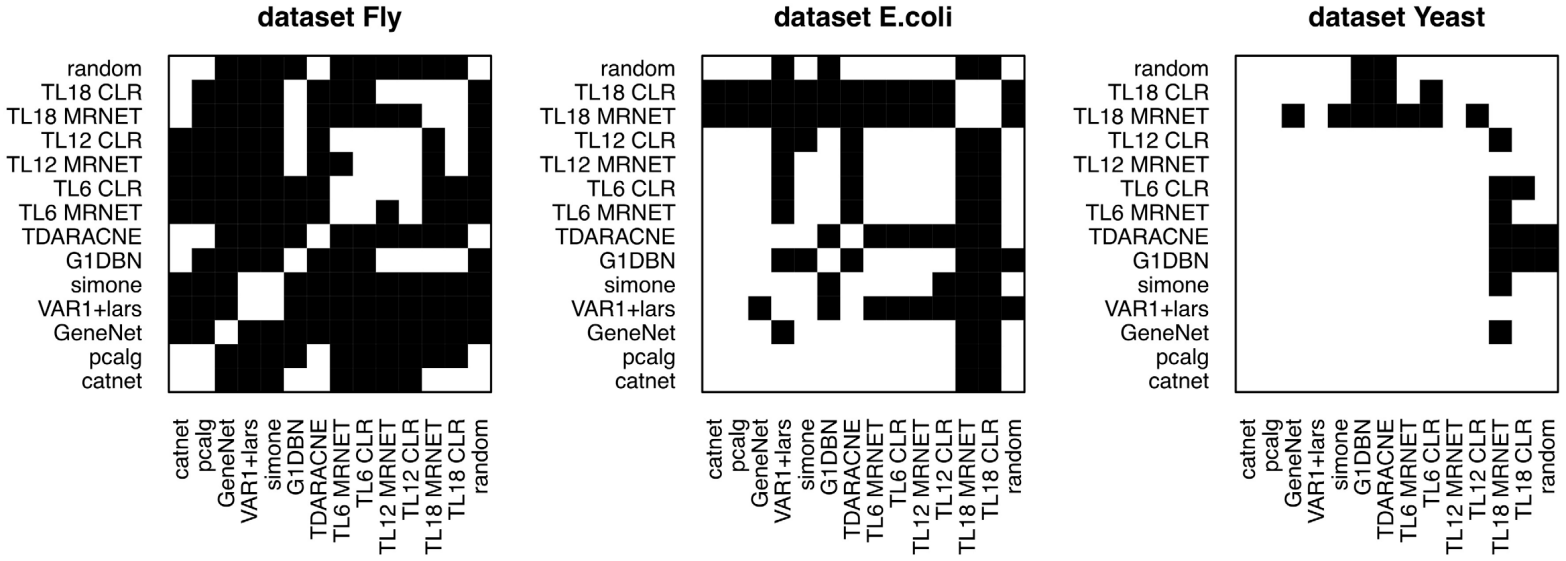
**Existence (black) or not (white) of a significant difference between the algorithms performance**.

The average AUPRC values for each algorithm and dataset can be seen in the Figure 2. The Figure 3 represents the existence (black), or not (white) of a significant difference between the performance of any two algorithms. All pairs of algorithms were subject to a paired t-test (two-sided, different variances) to test for a significant difference in their performance. The algorithms' AUPRC values were given as the input to the test and a difference was considered significant is the returned *p*-value was lower than 0.05. Of particular interest are the differences relative to the random ranking of interactions. Relative to the dataset Fly, dynamic models clearly outperform static models, which do not perform better than random. In the dataset *E.coli*, the best performers are the time lagged-MRNET and the time lagged-CLR when *l*_max_ is set to 18 time points (corresponding to 3 h). Fixed lag models and static models perform similarly, with only one method performing better than random (VAR1+lars). Relative to the dataset Yeast, the best performers are G1DBN and Time-Delay ARACNE, and are the only ones with a performance significantly better than random. As a control procedure, the ordering of the time points in the datasets was randomized, and the dynamic network inference methods were rerun (static models do not depend on the ordering of the samples). As expected, on all occasions the performance drops to the random level.

## 4. Discussion

Some points can be drawn from the results presented:

- The performance of some methods can be poor. On the dataset *E.coli* only three methods are better than random, and on the dataset Yeast there are only two. On the dataset Fly no static method performs better than random (the dynamic methods, on the contrary, perform well). This poor performance may be a result of the low number of samples of the datasets, or with the way the networks are generated and assessed, using gene regulatory interactions as a ground-truth that may not be adequate, or representative of the interactions that are regulating gene expression.
- The best performers on all datasets are dynamic models. This suggests that incorporating temporal information is beneficial to the inference of gene regulatory interactions. On all datasets, static models do not perform better than random. The fact that the assessed dynamic models are computationally simpler than the static algorithms (particularly the ones estimating Bayesian networks) is another reason to prefer dynamic models over static ones when inferring networks from time series.
- Most of the temporal models perform better on the dataset Fly than on the datasets Yeast and *E.coli* (see the comparison with random in Figure 3). This difference is possibly due to the temporal characteristics of the datasets (Fly is a 30-min interval dataset, of duration of 24 h; Yeast is a 5-min interval dataset, of duration 2 h). It seems natural that the gain in performance using dynamic models depends on the temporal characteristics of the dataset. On the dataset Fly, the dynamic performers also exhibit a significant difference between them. On the contrary, on the dataset Yeast, most of the models perform similarly (at the random level) and do not exhibit such difference.
- On the dataset Fly, the best performers are fixed lag methods. These methods directly estimate conditional dependencies, as opposed to the adaptive lag methods that only estimate pairwise dependencies. This aspect may play a role in the observed differences in performance.
- The performance of the adaptive lag models changes with the parameter *l*_max_. On the datasets Fly and Yeast there is a decrease in the performance of the time-lagged MRNET and CLR as *l*_max_ increases. On the dataset *E.coli*, on the contrary, there is a large performance boost when *l*_max_ is set to 18 time points.
- On the dataset Fly, a long time series where each time point corresponds to 30 min, setting *l*_max_ to too high values can be unrealistic (a lag of 18 time points corresponds to 9 h). If we estimate lagged dependencies over a long and unrealistic range of lags, it may happen that some genes that do not interact, are eventually found to be correlated at some lag. This may be the reason behind the decrease in performance, when *l*_max_ is set to high values.
- On the dataset *E.coli*, setting *l*_max_ to 18 time points greatly improves the performance. Here, 18 time points correspond to 3 h. This number may be an indication of the true range of values of gene interaction lags.
- Relative to the dataset Yeast, the performance decrease that is seen when setting *l*_max_ to 18 time points is likely to be a result of the fact that this dataset is composed of only 25 points. The number of samples used to estimate dependencies between genes varies from *n* to *n* − *l*_max_ where *n* is the number of samples in the dataset. On datasets of a low *n*, setting *l*_max_ to a high value may greatly reduce the number of samples used in the estimations, and if this number is too low, the variance of the algorithm increases, which causes the estimation of high correlations between genes that in reality do not interact. This may be happening in the case of the dataset Yeast, of 25 time points. When *l*_max_ is 90 min, the number of points used is only 7. If we compare with the dataset *E.coli*, when *l*_max_ is set to the maximum of 180 min, the number of samples used is still 14. When it comes to the dataset Fly, the number of samples used in the maximum *l*_max_, of 9 h, is 27.
- The performance of fixed lag models (lag being one time point) should be influenced by the interval length of the time series. These models should perform, relatively to static models, better on time series with interval lengths similar to the true lags of interactions. It can be seen that fixed lag models perform consistently better than static models on the dataset Fly. The same cannot be said regarding the other two datasets, where static and fixed lag models perform similarly. This may indicate that fixed lag, with lag equal to one, models are more appropriate to model time series with a temporal step relatively high, in the order of 30 min, than to model time series of shorter steps.

### 4.1. Analysis of lag distributions

**Figure 4:**
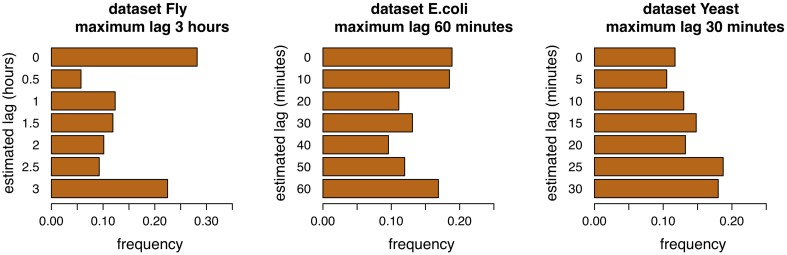
**Distribution of lags for the three datasets, maximum allowed lag is 6 time points**.

**Figure 5:**
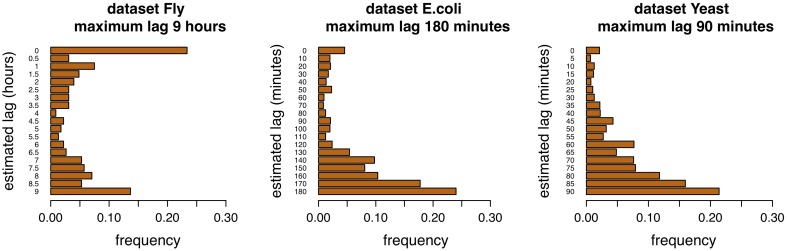
**Distribution of lags for the three datasets, maximum allowed lag is 18 time points**.

Adaptive lag algorithms are based on the estimation of lags between pairs of genes. These should reflect in some way the true lags of the interactions. The Figures 4, 5 show the distribution of the estimation of lags of true interactions, done by the algorithms time-lagged MRNET and time-lagged CLR, when the maximum allowed lag is set to 6 time points and 18 time points. There is a relatively high value of lags estimated to be 0, on all datasets. An explanation may be that a number of assumed interactions (taken from the regulatory interactions lists) are not correct, and that the respective genes, instead of one regulating the other, are in fact co-regulated. These results may provide insights on the temporal lag of gene interactions. Different interactions are possibly characterized by different lags, and these can depend on the biological function of the interacting genes. Also, it is likely that different species have different gene interaction lag times. On the dataset Fly, adaptive lag models see their performance decrease when *l*_max_ is set to 9 h. We suggest that this is due to the fact that, when setting *l*_max_ to such a high value, some interaction lags are estimated to be unrealistically high. This is confirmed in the Figure 5, when we see that there is a relatively large proportion of interaction lags estimated to be between 7 and 9 h. We also note a peak on estimated lag values between 1 and 2 h, that can be an indication of some of the true interaction lags. On the dataset *E.coli*, there is a large proportion of interaction lags estimated to be between 130 and 180 min. The fact that there is a great performance increase, when *l*_max_ is set to 180 min, suggests that maybe some interactions are characterized by these large lag values. However, it is possible that these high estimated lag values are a result of a decrease in the number of samples used to estimate the lagged dependencies. This phenomenon is certainly happening in the dataset Yeast, when the number of samples used to estimate dependencies reduces to 25% of the time series length (7 samples, or 30 min), when *l*_max_ is set to 90 min, and increasing the variance of the algorithm.

### 4.2. Study limitations

Only three gene expression datasets were used, each with its own distinct characteristics. Further validation of the results here presented should be made using other datasets, preferably with higher number of samples, as they become more available to bio-statisticians. The inference of regulatory interactions was done on networks of 5 genes. All things equal, the network inference models here presented will return lower AUPRC scores if the number of genes increases, and the ratio true edges/possible edges decreases - the inference task becomes more challenging. Network inference was assessed using interactions reported in the literature, which means some true interactions may be missing, and some reported interactions may be biologically inexistent in the used datasets.

## 5. Conclusion

Results obtained using three different datasets show that dynamic models perform better on the inference of gene regulatory interactions from time series, than static models such as Bayesian networks. This is explained by the inclusion of beneficial temporal information. Nevertheless, the overall performance of the assessed models is poor: only three and two models outperformed random in the *E.coli* and Yeast datasets, respectively. The differences in the results obtained in the datasets (a much higher performance variation in Fly, with most of the methods performing better than random) are likely due to the characteristics of the time series, such as the temporal interval. Regarding the dynamic models, the advantage of the considered fixed lag models is that they directly estimate conditional dependencies, instead of being based on pairwise dependencies, as the considered adaptive lag models are. On the other hand, the advantage of the adaptive lag models is that they can potentially infer interactions characterized by higher and variable lags. Their performance depends on the maximum allowed lag, *l*_max_, and care should be taken when defining this parameter: if it is set to an unrealistic high value, in the range of many hours, eventually interactions will be estimated at that range, hurting the network inference performance (we argue that this is seen in the results regarding the dataset Fly). If *l*_max_ is set to be equal to a high fraction of the length of the time series, lagged dependencies between genes will be estimated with a small number of samples, increasing the variance of the algorithm and decreasing its performance (this is seen in the results regarding the dataset Yeast). Relative to the lag of regulatory gene interactions, the fact that lag-one models (the fixed lag models) perform, compared with static models, better on a dataset with a temporal interval of 30 min than in datasets with lower temporal intervals (10 and 5 min) suggests that the range of lags of gene interactions is likely to be closer to 30 min than to 10 or 5 min. The experimental results also suggest that there may exist gene interactions characterized by a longer lag, in the order of a couple of hours. As a general set of rules, we conclude from the experiments here reported that dynamic methods should be used to predict interactions in time series; fixed lag methods (estimating conditional dependencies) should be used when the interval scale is high (30 min to hours); adaptive lag methods should be used when the maximum allowed lag is set to high values (order of a couple of hours), and, in order to prevent an excessive algorithm variance, the number of samples minus the maximum allowed lag is still high (the results obtained on the *E.coli* dataset suggest this value to be at least 14 samples).

## Author contributions

Miguel Lopes designed and implemented the experimental run, and contributed to the writing of the paper. Gianluca Bontempi supervised the study and contributed to the writing of the paper.

### Conflict of interest statement

The authors declare that the research was conducted in the absence of any commercial or financial relationships that could be construed as a potential conflict of interest.
